# No Evidence of XMRV or MuLV Sequences in Prostate Cancer, Diffuse Large B-Cell Lymphoma, or the UK Blood Donor Population

**DOI:** 10.1155/2011/782353

**Published:** 2011-06-09

**Authors:** Mark James Robinson, Philip William Tuke, Otto Erlwein, Kate I. Tettmar, Steve Kaye, Kikkeri N. Naresh, Anup Patel, Marjorie M. Walker, Takahiro Kimura, Ganesh Gopalakrishnan, Richard S. Tedder, Myra O. McClure

**Affiliations:** ^1^Section of Infectious Diseases, Jefferiss Research Trust Laboratories, Imperial College London, St Mary's Campus, London W2 1PG, UK; ^2^Transfusion Microbiology R&D, National Transfusion Microbiology Laboratories, NHS Blood and Transplant, Colindale, London NW9 5BG, UK; ^3^Centre for Pathology, Hammersmith Hospital, Imperial College Health Network NHS Trust, 115 1st Floor, L Block, London W12 0HS, UK; ^4^Urology Department, St Mary's Hospital, Imperial College Healthcare NHS Trust, London W2 1NY, UK; ^5^Histopathology Department, St Mary's Hospital, Imperial College London, London W2 1NY, UK; ^6^Department of Urology, The Jikei University School of Medicine, 3-25-8, Nishi-Shinbashi, Minato-ku, Toyko 105-8461, Japan; ^7^Consultant Urologist, Vedanayagam Hospital, RS Puram, Coimbatore-2 641002, India; ^8^Blood Borne Viruses Unit, Viralus Reference Department, Centre for Infections, Health Protection Agency, 61 Colindale Avenue, London NW9 5EQ, UK

## Abstract

Xenotropic murine leukaemia virus-related virus (XMRV) is a recently described retrovirus which has been claimed to infect humans and cause associated pathology. Initially identified in the US in patients with prostate cancer and subsequently in patients with chronic fatigue syndrome, doubt now exists that XMRV is a human pathogen. We studied the prevalence of genetic sequences of XMRV and related MuLV sequences in human prostate cancer, from B cell lymphoma patients and from UK blood donors. Nucleic acid was extracted from fresh prostate tissue biopsies, formalin-fixed paraffin-embedded (FFPE) prostate tissue and FFPE B-cell lymphoma. The presence of XMRV-specific LTR or MuLV generic *gag*-like sequences was investigated by nested PCR. To control for mouse DNA contamination, a PCR that detected intracisternal A-type particle (IAP) sequences was included. In addition, DNA and RNA were extracted from whole blood taken from UK blood donors and screened for XMRV sequences by real-time PCR. XMRV or MuLV-like sequences were not amplified from tissue samples. Occasionally MuLV *gag* and XMRV-LTR sequences were amplified from Indian prostate cancer samples, but were always detected in conjunction with contaminating murine genomic DNA. We found no evidence of XMRV or MuLV infection in the UK blood donors.

## 1. Introduction

In 2006, a new gammaretrovirus, xenotropic murine leukaemia virus-related virus (XMRV), was discovered by the Virochip analysis in prostate cancer tissue from patients homozygous for an RNase L mutation [1]. In these patients, the innate antiviral defence RNase L pathway is defective; hence, these patients are likely to be susceptible to viral infection and a population more likely to find a novel virus with disease association in. When a second US study found that 6% of all prostate cancer patients, independent of RNase L mutations, were infected with the virus, thus broadening the population at risk [2], interest in XMRV intensified. However, subsequent studies from the USA [3, 4] and all European studies [5–7] failed to confirm the presence of XMRV in prostate tissue. More recently it has been suggested that XMRV detection in prostate tissue in the US could be related to the specificity and conditions of the PCR used [8]. 

In 2009, Lombardi and colleagues reported the presence of XMRV proviral DNA in peripheral blood leucocytes from 3.7% of healthy controls and 67% of patients with chronic fatigue syndrome (CFS) [9]. The detection rate by PCR amplification of XMRV proviral DNA subsequently reduced the estimated CFS prevalence to 7%, with the explanation that RNA extraction and cDNA synthesis had been required to achieve the 67% prevalence originally reported [10]. Lo and colleagues (2010) using predominantly archival material from patients with CFS detected a high prevalence (86.5%) of pMuLVs. These are similar to, but constitute a different group from, the xenotropic endogenous MuLVs to which XMRV belongs [11]. However, questions were raised about how these data were generated [12], and a number of other studies have failed to demonstrate a link between XMRV or pMuLV infection and CFS [13–19]. 

The causes of B-cell lymphoma are not fully understood [20], but the clinical and epidemiological characteristics are suggestive of the involvement of an infectious agent [21]. Several viruses [22, 23] have been linked to the risk of B-cell lymphoma, most notably EBV [24–26], and retroviruses are implicated in animal leukaemias. Retroviral integration could cause somatic DNA changes leading to clonal expansion of B cells resulting in leukaemia as has been previously described for adult T-cell leukaemia (ATL) and HTLV-1 [27].

The geographical discrepancy of XMRV and pMuLV prevalence remains unexplained. To explore this further, we have tested a variety of tissues from diverse populations; prostate cancer (PC) formalin-fixed paraffin-embedded (FFPE) tissue from Japan and India, fresh prostate tissue samples received from the Urology Clinic at St Mary's Hospital, London, and peripheral blood from English blood donors. 

A series of recent papers [28–31] have demonstrated the ease with which specimens can be contaminated with murine DNA sequences. To control for this, all tissue specimens were tested by PCR specific for intracisternal A particle (IAP), a retrotransposon present in multiple copies (~1000) within the mouse genome [32]. 

## 2. Methods and Materials

### 2.1. Samples and Nucleic Acid Isolation

Prostate biopsies were collected from 55 patients admitted to the Urology Department, St. Mary's Hospital, London, UK to undergo routine biopsy for prostate cancer screening. All patients gave written informed consent for their tissue to be banked for the purposes of research (ethics number 99/CCC/166, August 1999). The DNA was extracted using the QIAamp DNA mini kit (Qiagen, Crawley, UK) following the manufacturer's instructions.

B-cell lymphoma samples were provided by Professor Kikkiri Naresh, Centre for Pathology, Hammersmith Hospital, London, UK. The DNA from 10 Diffuse Large B-cell Lymphoma (DLBLC) patients was extracted from FFPE tissues of lymph nodal or extranodal diffuse large B-cell lymphoma using the DNeasy Blood & Tissue Kit (Qiagen). Briefly, two 15 *μ*m sections were cut and transferred to 1.5 mL Eppendorf tubes. Blades were changed between samples to avoid cross-contamination. Sections were deparaffinised with xylene and ethanol, rehydrated, and incubated with proteinase K and lysis buffer in a shaking water bath at 55°C overnight and the extraction was completed according to the manufacturer's instructions.

Twenty FFPE prostate specimens including 10 prostate cancer (PC) and 10 benign prostatic hyperplasia (BPH) samples were supplied by Professor Ganesh Golpalakrishnan of Vedanayagam Hospital, RS Puram, Coimbatore, India and sixteen specimens from Dr. Takahiro Kimura of the Department of Urology, The Jikei University School of Medicine, Japan. From the Indian blocks, two 10 *μ*M sections were extracted with the QIAamp DNA FFPE tissue kit (Qiagen), according to the manufacturer's instructions. The Japanese samples were provided presliced on glass slides. 

Random anonymous whole-blood samples were obtained from the Donation Testing Department at the National Health Service Blood and Transplant (NHSBT) Centre at Colindale, London, UK. Plasma minipools were similarly obtained from NHSBT. All blood and plasma samples were extracted on a Qiagen MDx Biorobot and eluted with 80 *μ*L of Qiagen buffer AVE.

### 2.2. XMRV, MuLV, and Control Nested PCR

Samples were tested for the presence of XMRV and MuLV proviral DNA using nested PCR, as described previously [14]. Briefly, we used a set of primers that encompasses the 24 bp deletion in the XMRV *gag* leader region, originally described to distinguish XMRV as a new human virus, along with a second set of primers reflecting a sequence conserved amongst most MuLVs. The positive control for the XMRV and MuLV PCRs was plasmid VP62 [1]. The PCR method has been shown to be sensitive enough to pick up one copy of XMRV VP62 plasmid in a background of 500 ng DNA [28]. As a control for sample addition and PCR inhibition, primers to the human beta-globin (hBG) gene were used. DNA extracted from LNCaP (human prostate cancer cells) was used as a positive control for human beta globin. To control for contamination of samples with murine DNA, primers specific to mouse IAP were used as described previously [28]. The positive control for IAP was DNA from the McCoy cell (murine fibroblast cells, ECAAC 90010305). In all PCRs, at least 6 “no template” controls were set up. All PCR products were visualised on Ethidium Bromide-stained 2% agarose gels.

### 2.3. XMRV, MuLV, and Control Real-Time PCRs for Blood Donor Studies

Real-time PCR was performed as detailed in Table 1. For the proviral DNA analysis, 10 *μ*L of the nucleic acid extract were analysed separately in three individual quantitative PCRs (Q-PCRs).

#### 2.3.1. XMRV Q-PCR and Internal Control

Samples were tested by Q-PCR for XMRV, as described by McCormick et al. [33] and modified as detailed in Table 1. In a Q-PCR to control for the extraction efficiency and amplification inhibition coextracted soil-borne cereal mosaic virus (SBCMV) plasmid DNA was used, (5.4 × 10^6^ copies were added to the 33 mL of Qiagen lysis buffer AL used for extracting 96 samples on the MDx Biorobot). This reaction was as described by Ratti et al. [34]. The primer sequences for this reaction were *SBCWMVCPF* (5′-CAC TCA GGA CGG TGA CGA GAT-3′), SBCWMVCPR (5′-GTG ATA CTG TGA GTC TGG TGA TGA TTT-3′) and probe *SBWMV237Fa* (5′ JOE-TTT TGT GAC CTT GGA GGT GAG GCA GTT ATG-BHQ1-3′).

#### 2.3.2. Q-PCR for Quantification of Human DNA

The input of human DNA in each extract was measured by a Q-PCR for the Pyruvate dehydrogenase (PDH) gene. Primers used *PDH Taq 1 *(5′-TGA AAG TTA TAC AAA ATT GAG GTC ACT GTT-3′), *PDH Taq 2* (5′- TCC ACA GCC CTC GAC TAA CC -3′) with probe *PDHP* (5′-VIC-CCC CCA GAT ACA CTT AAG GGA TCA ACT CTT AAT TGT-Tamra-3′). Positive control for this reaction was a dilution series of human male DNA (Applied Biosystems, Warrington, UK, Catalogue no. 4312660). The XMRV Q-PCR results were validated when the PDH threshold cycle (Ct) value was greater than the mean Ct minus 3 SD, and the SBCMV control was greater than the mean Ct minus 2 SD. Samples invalid on either control were excluded from the analysis.

### 2.4. Detection of Gag Sequences by Nested PCR in Blood Donors

Nuclease-free water (Severn Biotech, Kidderminster, UK) was used throughout for the cDNA and PCR mix preparations and as no-template controls. Nucleic acid extracts were tested by nested PCR using the *gag* primers as described by Lombardi et al. [9] and Lo et al. [11], but using Applied Biosystems Taq Gold LD PCR enzyme (Table 1) to overcome the problem of false positives that have arisen from the use of Invitrogen Taq Polymerase [30]. 

### 2.5. QRT-PCR Amplification of XMRV/pMuLV in Blood Donors

An XMRV/pMuLV *gag* QRT-PCR assay described by Lo and colleagues [11] but modified to detect the pMuLVs was used to test nucleic acid from whole blood, plasma, and from plasma minipools. Further details of all QPCR and QRT-PCR reactions are listed in Table 1. The primers for this reaction were *F3* (5′-ACC GTT TGT CTC TCC TAA AC-3′) and *R4* (5′-AGG GTA AAG GGC AGA TCG-3′), with probe *P2* (5′-Fam-CCG ACA GCT CCC GTC CTC CCG-Tamra-3′). Nuclease-free water (Severn Biotech) was used throughout for the RT-PCR mix preparations and as no template controls. RT-PCR was performed in a total volume of 50 *μ*L, containing 1x Qiagen QuantiTect RT-PCR buffer and primers, and probes as detailed in Table 1. Synthesis conditions were 50°C for 30 mins, followed by 95°C for 15 mins and 45 cycles of 95°C for 15 secs 60°C for 1 min. Twenty *μ*l of nucleic acid was analysed in a QRT-PCR which multiplexed the XMRV/pMuLV TaqMan with the internal control TaqMan reaction (Brome mosaic virus (BMV)) [35]. The BMV RNA was added to the Qiagen AL lysis buffer and co-extracted with the sample. A sample was valid if the BMV Ct value was greater than the mean Ct minus 2 SD. Samples invalid on the BMV control were excluded from the analysis. The sensitivity of this QRT-PCR was determined as 150 RNA copies/mL (75 viral particles/mL) by calculation from the observed frequency of negatives using the Poisson distribution.

## 3. Results

### 3.1. XMRV Detection in Tissue Samples by Nested PCR

**Figure 1:**
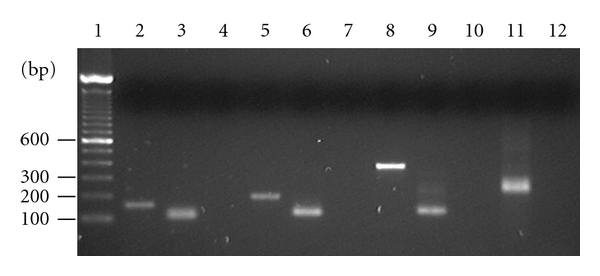
lane 1: MWM; lanes 2–4: *β*-globin PCR on LNCaP DNA template 1st round product, 2nd round product, and no-template control; lanes 5–7: XMRV LTR PCR on VP62 plasmid DNA template 1st round product, 2nd round product, and no-template control; lanes 8–10: MuLV *gag* PCR on VP62 plasmid DNA template 1st round product, 2nd round product, and no-template control; lanes 11-12: IAP PCR on McCoy cell DNA template and no-template control.

A representative stained gel following nested PCR is shown in Figure 1. For routine analysis, 0.11 pg of plasmid DNA (representing approximately 7000 copies/PCR) was used as positive control for XMRV and MuLV. All samples were positive for hBG sequences by PCR. The sensitivity of the IAP PCR has been shown previously to detect as little as 0.0011 pg DNA in a background of 500 ng DNA [28]. The results are summarised in Table 2(a). No evidence of XMRV or MuLV was found in any of the FFPE prostate tissue samples from Japan or the fresh prostate tissues from the UK. Of the 20 Indian samples, four (20%) produced a PCR signal with the MuLV *gag *primers (three prostate cancer, one benign prostatic hyperplasmia) and of these, 2/4 were positive with XMRV LTR primers (both prostate cancer). The IAP PCR was applied to the same samples to see if the positive signal was due to mouse DNA contamination. All MuLV/XMRV amplification was concordant with IAP amplification, except for one prostate cancer sample which was positive for IAP without MuLV/XMRV amplification. Confirmation of murine DNA contamination was achieved using PCR primers specific to mouse mitochondrial DNA (mtDNA). Although this PCR has been shown to be less sensitive than IAP PCR [28], 2/20 of the Indian samples (one prostate cancer, one benign prostatic hyperplasia) were positive for mtDNA. In both of these samples, IAP and MuLV *gag *sequences were amplified. Additionally, one was positive for XMRV (detailed in Table 2(b)).

No evidence of MuLV or XMRV sequences was discovered in the DLBCL samples and none of the DCBCL samples gave an IAP specific product.

### 3.2. XMRV Detection in Whole Blood by Real-Time PCR

XMRV proviral DNA was not amplified from whole-blood extracts derived from 540 donors. The average DNA input for each amplification was 93,000 cells (approx 0.56 *μ*g). Detection of XMRV/MuLV RNA was undertaken on a further 600 donors and 400 plasma minipools, derived from 19,200 individual donations. All samples tested negative for XMRV and MuLV sequences.

## 4. Discussion

Using highly sensitive PCRs with primers that detect XMRV and primers that detect MuLV-like sequences, no proviral DNA was detected in any of the prostate cancer samples independently of murine DNA contamination. This served to confirm our previous studies in which FFPE prostate tissue was tested and XMRV/MuLV sequences failed to be amplified [28]. Here we have added further data to show that no XMRV or MuLV-like sequences can be detected in fresh UK prostate tissue or in prostate cancer samples collected from Japan. Samples from India showed evidence of MuLV and XMRV sequences when viral genomic sequences were amplified by nested PCR. However, this was concordant with murine genomic DNA contamination detected using primers to IAP. IAPs are retrotransposons present at the level of around 1000 copies per mouse genome [30]. Thus, IAP PCR represents a highly sensitive detection method for murine DNA. Although the sample size was small (*n* = 10), we found no evidence to suggest that XMRV might be involved in other cancers, such as diffuse large B-cell lymphoma. 

 It was reported last year that XMRV had been detected in greater than 60% of 50 samples from English blood donors [36]. In contrast, we found no evidence of XMRV or pMuLV in any of 540 whole-blood samples from unselected NHSBT donors nor were we able to detect MuLV-like sequences in either the DNA from whole blood or cDNA prepared from the plasma minipools from donors in England. There are three possible explanations for this. Firstly, there are no MuLV infections in blood donors in England. Secondly, there are MuLV infections, but that the assays used failed to detect them, either due to sensitivity or sequence variation. Thirdly, there are MuLV infections, but the prevalence is too low to be detected in the sample sizes tested.

Research into the presence of MuLVs in the human population is contentious, given discrepant findings [37–39]. Contamination from sequences contained in apparently XMRV-positive samples, amplified products, or plasmids has been suggested as a reason for the finding of MuLVs in human samples [30, 40]. A study of XMRV in patients with CFS or chronic immunomodulatory conditions, using Invitrogen Platinum Taq (IPT), reported a *gag* sequence with >99% homology to a mouse endogenous retrovirus [19]. This was designated as contamination, although the paper failed to speculate on the source of this sequence. Sato and colleagues (2010) recently reported finding predominantly RNA sequences, related to a pMuLV, in IPT containing reagents [30]. Another study concluded that the detection of MuLV-related sequences in human samples could be due to contamination with mouse DNA, most likely contained in various laboratory reagents [29]. We have demonstrated that murine sequences can be present in prostate sections, resulting in false positive detection of XMRV [28]. A phylogenetic overview concluded that the proviral sequences present in the genome of 22Rv1 cell line were ancestral to the published XMRV sequences [31]; finally, it has been shown that the mapping of integration sites of XMRV in prostate cancer tissues, thought to unequivocally confirm the existence of XMRV in clinical samples, was at least partially contaminant derived [41], further emphasising the ease with which contamination can occur.

The sources of contamination are still to be fully elucidated. However, given that most retroviral laboratories have worked with MuLV or MuLV-derived vector systems, or at least used murine reagents, it is essential that sufficient appropriate controls are included in all PCRs. 

The absence of MuLVs from all the samples analysed in this study, where there was no concomitant detection of murine genomic sequences, adds weight to the growing body of data questioning the evidence for murine retrovirus infection of humans [42]. It is always challenging to prove a negative result, but it is likely that XMRV will be added to the long list of RNA rumour viruses [43].

##  Conflict of Interests

The authors declare no conflict of interests.

## Figures and Tables

**Table 1:** Details of PCRs used to test blood sample.

Sample tested	PCR	Target	Primers and probes	Cycles (*N*)	Reagents
540 DNAs from whole blood	XMRV Taq Man	gag	XMRV Probe, F, R	60	Qiagen QuantiTect Probe kit
540 DNAs from whole blood	SBCMV Taq Man	SBCMV plasmid	SBCWMVCPF, SBCWMVCPR, SBWMV237F	45	ABgene ABsolute QPCR ROX mastermix
540 DNAs from whole blood	PDH Taq Man	PDH human gene	PDH Probe, F, R	45	ABgene ABsolute QPCR ROX mastermix
600 NAs from whole blood 400 NAs from plasma minipools	XMRV/pMuLV RT Taq Man with BMV RT Taq Man	gag BMV	P2, F3, R4 BMV Probe, F, R	45	Qiagen QuantiTect Probe RT-PCR kit

The TaqMan assay conditions were 15 min at 95°C (15 secs 95°C, 1 min 60°C) × *N* cycles. 400 nM concentrations of primers, and 200 nM probes were used in all the TaqMan assays with the exception of the CDC MuLV Taq Man where the concentration of each probe was 100 nM and the PDH TaqMan where the primer concentrations were 50 nM.

**Table:** (a) Amplification from fresh and FFPE tissues by nested PCR

	Fresh prostate tissue	Japan samples	Indian samples	LCBCL samples
Number of cancerous samples	16/55	16	10/20	10
Number of noncancerous samples (unknown status)	18/55 (21/55)	0	10/20	0
Mean age (range)	unknown	unknown	72 (62–85)	43 (27–83)
Beta globin +	55/55	16/16	20/20	10/10
XMRV +	0	0	2/20	0
MuLV +	0	0	4/20	0
IAP +	0	0	5/20	0
mtDNA +	nd	nd	2/10	nd

**Table:** (b) Specific PCR results from Indian samples

Indian sample number	Cancer status	PCR result using specific primers				
		*β*-globin	IAP	mtDNA	MLV *gag *	XMRV LTR

6489c/10	cancer	+	+	+	+	+
5383c/10	cancer	+	+	−	+	+
5406a3/10	cancer	+	+	−	−	−
2896c/10	BPH	+	+	+	+	−
5349c/10	cancer	+	+	−	+	−
